# Charles S. Brower

**Published:** 1913-04

**Authors:** 


Charles S. Brower, P. & S., Baltimore, 1880, died in Kingston, N. Y., December 15. He was a practitioner of Phoenicia, N. Y.

